# Supplementary material for: Empirical methods for controlling false positives and estimating confidence in ChIP-Seq peaks
Source: BMC Bioinformatics. 2008 Dec 5;9:523. doi: 10.1186/1471-2105-9-523 (PMC2628906; doi:10.1186/1471-2105-9-523)
Supplement: Additional File 1 — A variety of html documents from the USeq web site detailing the available applications, their best usage, output file type descriptions, command line menus, etc. [file 1471-2105-9-523-S1.zip › USeqUserGuides/JavaDoc.zip!/JavaDoc/cgar/beans/AnalysisBean.html]

AnalysisBean


|  |  |  |
| --- | --- | --- |
| **Overview** \| **Package** \| **Class** \| **Use** \| **Tree** \| **Deprecated** \| **Index** \| **Help** | |  |
| PREV CLASS   **NEXT CLASS** | **FRAMES**    **NO FRAMES**     **All Classes** |  |
| SUMMARY: NESTED \| FIELD \| CONSTR \| METHOD | DETAIL: FIELD \| CONSTR \| METHOD |  |


---


## cgar.beans Class AnalysisBean

```
java.lang.Object
  cgar.beans.AnalysisBean
```

---

``` public class AnalysisBean extends java.lang.Object ```

---

**Constructor Summary**

`AnalysisBean()`


| **Method Summary** | |
| --- | --- |
| `java.lang.String` | `getDate()` |
| `java.lang.String` | `getDescription()` |
| `int` | `getGenomeId()` |
| `int` | `getId()` |
| `int` | `getLabGroupId()` |
| `java.lang.String` | `getName()` |
| `int` | `getProtocolId()` |
| `int` | `getProtocolNotes()` |
| `int` | `getUserId()` |
| `int` | `getVisibilityId()` |
| `void` | `setDate(java.lang.String date)` |
| `void` | `setDescription(java.lang.String description)` |
| `void` | `setGenomeId(int genomeId)` |
| `void` | `setId(int id)` |
| `void` | `setLabGroupId(int labGroupId)` |
| `void` | `setName(java.lang.String name)` |
| `void` | `setProtocolId(int protocolId)` |
| `void` | `setProtocolNotes(int protocolNotes)` |
| `void` | `setUserId(int userId)` |
| `void` | `setVisibilityId(int visibilityId)` |

**Methods inherited from class java.lang.Object**

`equals, getClass, hashCode, notify, notifyAll, toString, wait, wait, wait`

**Constructor Detail**

### AnalysisBean

```
public AnalysisBean()
```


**Method Detail**

### getDate

```
public java.lang.String getDate()
```

---


### setDate

```
public void setDate(java.lang.String date)
```

---


### getDescription

```
public java.lang.String getDescription()
```

---


### setDescription

```
public void setDescription(java.lang.String description)
```

---


### getGenomeId

```
public int getGenomeId()
```

---


### setGenomeId

```
public void setGenomeId(int genomeId)
```

---


### getId

```
public int getId()
```

---


### setId

```
public void setId(int id)
```

---


### getLabGroupId

```
public int getLabGroupId()
```

---


### setLabGroupId

```
public void setLabGroupId(int labGroupId)
```

---


### getName

```
public java.lang.String getName()
```

---


### setName

```
public void setName(java.lang.String name)
```

---


### getProtocolId

```
public int getProtocolId()
```

---


### setProtocolId

```
public void setProtocolId(int protocolId)
```

---


### getProtocolNotes

```
public int getProtocolNotes()
```

---


### setProtocolNotes

```
public void setProtocolNotes(int protocolNotes)
```

---


### getUserId

```
public int getUserId()
```

---


### setUserId

```
public void setUserId(int userId)
```

---


### getVisibilityId

```
public int getVisibilityId()
```

---


### setVisibilityId

```
public void setVisibilityId(int visibilityId)
```


---


|  |  |  |
| --- | --- | --- |
| **Overview** \| **Package** \| **Class** \| **Use** \| **Tree** \| **Deprecated** \| **Index** \| **Help** | |  |
| PREV CLASS   **NEXT CLASS** | **FRAMES**    **NO FRAMES**     **All Classes** |  |
| SUMMARY: NESTED \| FIELD \| CONSTR \| METHOD | DETAIL: FIELD \| CONSTR \| METHOD |  |


---
